# Levistolide A Inhibits PEDV Replication via Inducing ROS Generation

**DOI:** 10.3390/v14020258

**Published:** 2022-01-27

**Authors:** Wei Zeng, Jingping Ren, Zhonghua Li, Changsheng Jiang, Qi Sun, Chang Li, Wan Li, Wentao Li, Qigai He

**Affiliations:** 1 State Key Laboratory of Agricultural Microbiology, College of Veterinary Medicine, Huazhong Agricultural University, Wuhan 430070, China; aiyouwei@webmail.hzau.edu.cn (W.Z.); rjp@webmail.hzau.edu.cn (J.R.); changshengjiang@webmail.hzau.edu.cn (C.J.); sunqi1019@webmail.hzau.edu.cn (Q.S.); lichang1113@webmail.hzau.edu.cn (C.L.); liwan@webmail.hzau.edu.cn (W.L.); wentao@mail.hzau.edu.cn (W.L.); 2 The Cooperative Innovation Center for Sustainable Pig Production, Huazhong Agricultural University, Wuhan 430070, China; 3 Hubei Key Laboratory of Animal Nutrition and Feed Science, School of Animal Science and Nutritional Engineering, Wuhan Polytechnic University, Wuhan 430023, China; yuxincangqiong@163.com

**Keywords:** porcine epidemic diarrhea virus, Levistolide A, reactive oxygen species, antiviral

## Abstract

Porcine epidemic diarrhea virus (PEDV) variant strains adversely affect the production of pigs globally. Vaccines derived from PEDV traditional strains impart less protection against the variant strains. Moreover, sequence diversity among different PEDV variant strains is also complicated. This necessitates developing alternative antiviral strategies for defending against PEDV. This study explored a natural product, Levistolide A (LA), to possess antiviral activity against PEDV. LA was found to suppress PEDV replication in a dose-dependent manner. And the inhibitory effect of LA against PEDV was maintained in the course of time. In terms of viral RNA and protein production, LA also showed a strong inhibitory effect. In addition, LA was indicated to inhibit PEDV from attaching to the cellular membrane or penetrating the cells. Further study revealed that LA can induce the generation of reactive oxygen species (ROS), and the corresponding inhibitor, NAC, was found to antagonize the effect of LA on inhibiting PEDV replication. This illustrated that the LA-induced ROS generation played an important role in its anti-PEDV activity. LA was also identified to stimulate ER stress, which is an important consequence of ROS production and was proven to be able to inhibit PEDV replication. To conclude, this study revealed that LA can inhibit PEDV replication via inducing ROS generation.

## 1. Introduction

Porcine epidemic diarrhea virus (PEDV) primarily causes the neonatal piglet to develop severe watery diarrhea [1]. It is a positive-sense, single-stranded RNA togavirus, belonging to the *Coronaviridae* family, *Alphacoronavirus* genus [2]. There are at least seven open reading frames (ORFs) between the 5′untranslated region (5′UTR) and 3′UTR in the PEDV genome, including ORF1a, ORF1b, spike(S) glycoprotein gene, ORF3 hypothetical protein gene, envelope(E) protein gene, membrane(M) glycoprotein gene, and nucleocapsid(N) protein gene [3]. PEDV infection leads to a disease called porcine epidemic diarrhea (PED), which is characterized by vomiting, dehydration, anorexia, watery diarrhea, and weight loss in piglets. PED was first identified in the UK in the 1970s, it was then subsequently reported in other countries, including Belgium, Germany, the Netherlands, France, and China [4,5]. In 2010, PEDV variant strains were identified and were found to spread rapidly to different countries [6]. The neonatal piglets infected with PEDV variant strains showed almost 100% mortality [7]. PEDV variant strains have adversely affected the production of pigs globally and have incurred considerable economic losses [6,8,9].

*Ligusticum chuanxiong* has been prevalently applied in traditional Chinese medicine prescriptions. It has an important active component called Levistolide A (LA), which is polymerized by two molecular (Z)-ligustilide [10]. LA possesses multiple biological activities. It was reported that LA can suppress cancer cells through various approaches, such as cell cycle arrest and induction of cell apoptosis [11,12]. Furthermore, this polymer can enhance the efficacy of other anti-tumor drugs. It has been reported that LA possessed the ability to intensify doxorubicin-induced apoptosis of k562/dox cells [13]. In addition, it has been revealed to exhibit a reverse effect on P-glycoprotein-mediated drug resistance in human breast carcinoma cells [11]. Moreover, LA was identified to be potent for curing Alzheimer’s disease. A study revealed that LA could effectively attenuate Alzheimer’s pathology by activating the PPARγ pathway [14]. In terms of defending against infectious diseases, the component of LA was found to be partly active in an antiviral screen [15].

This study explored the precise effect of LA on PEDV replication. We demonstrated that LA exhibited antiviral activity against PEDV in a dose-dependent manner and this effect was maintained in the course of time. Apart from these, incubation with LA was indicated to potently suppress PEDV RNA and protein production. Moreover, LA was identified to inhibit PEDV from attaching to the cellular membrane or penetrating the cells. We further stated that LA led to the generation of reactive oxygen species (ROS) for suppressing PEDV propagation and the consequent endoplasmic reticulum stress might be important for this process.

## 2. Materials and Methods

### 2.1. Viruses, Cells, Antibodies, Chemicals, and Other Reagents

Vero cells (CCL81) were cultured in Dulbecco’s Modified Eagle Medium (DMEM) (Gibco, Waltham, MA, USA) supplemented with 10% FBS (NEWZERUM, Christchurch, New Zealand) and incubated at 37 °C with 5% CO_2_. LLC-PK1 cells were cultured in DMEM supplemented with 10% FBS and incubated at 37 °C with 5% CO_2_. PEDV strain DR13 (GenBank accession No. JQ023161) and PEDV strain CV777 (GenBank accession No. AF353511.1) were cultured in Vero cells in DMEM. PEDV strain YN15 (GenBank accession No. KT021228.1) and PEDV strain GDU (GenBank accession No. KU985230) were cultured in Vero cells in DMEM containing trypsin (Gibco, Waltham, MA, USA) at a dose of 8 µg/mL. Mouse anti-PEDV S protein and mouse anti-PEDV N protein monoclonal antibodies were generated in our laboratory [16]. Rabbit anti-GRP78 polyclonal antibody, Rabbit anti-β-Actin Monoclonal antibody, HRP goat anti-mouse IgG, and HRP goat anti-rabbit IgG were purchased from ABclonal (Wuhan, China). Alexa Fluor 488 Donkey anti Mouse IgG were purchased from AntGene (Wuhan, China). Levistolide A and Tunicamycin were purchased from MedChemExpress (Shanghai, China). DAPI and N-Acetylcysteine were purchased from Beyotime Biotechnology (Shanghai, China).

### 2.2. Cell Counting Kit-8 Assay

The cell viability was measured through the Cell Counting kit-8 (CCK8) (Beyotime, Shanghai, China) assay according to the instructions mentioned. Cells were seeded to 96-well plates and rinsed three times with PBS when the cells had reached approximately 100% confluent. After a 48 h incubation, the cells were cultured in DMEM containing the test chemicals at designed concentrations for 48 h. Subsequently, the cells were washed three times with PBS, then the cultured supernatant in each well was replaced with 110 µL DMEM containing 9.09% CCK-8 reagent, followed by culturing in the dark for 2 h. After gentle shaking, the optical density (OD) 450 of each well was measured using a Multimode Plate Reader (VICTOR Nivo, PerkinElmer, Waltham, MA, USA). Cell viability value was calculated according to the equation: the cell viability value = (As − Ab) ÷ (Ac − Ab) × 100%, where, As: after incubation with CCK-8 solution, the OD450 values of LA or DMSO treated wells; Ab: after incubation with CCK-8 solution, the OD450 values of wells without cells; Ac: after incubation with CCK-8 solution, the OD450 values of mock treated-wells.

### 2.3. TCID_50_ Assay

The samples were subjected to 3 cycles of freezing and thawing, followed by centrifugation at 12,000× *g* for 10 min at 4 °C. The supernatant was collected and serially diluted (100 µL supernatant in 900 µL virus culture medium) 8 times. Vero cells were seeded to 96-well plates and rinsed three times with PBS when the cells had reached approximately 100% confluent. The medium in every well of each group (each group contained 8 wells) was replaced with 100 µL diluent at the corresponding dilution. The cells were cultured for 72 h and the TCID_50_ value of each sample was calculated according to the Reed–Muench method established by L. J. Reed and H. Muench.

### 2.4. Indirect Immunofluorescence Assay

Cells were seed to 24-well plates and rinsed three times with PBS when the cells had reached approximately 100% confluence. The culture medium was replaced with the designed solution and the cells were continuously cultured for the indicated time. Then, the cells were fixed with 200 µL paraformaldehyde (4%) for 20 min at 25 °C. After rinsing three times with PBS, the cells were permeabilized with 0.1% Triton X-100 for 10 min at 25 °C. Subsequently, the cells were rinsed three times with PBS, and then blocked with PBS solution containing 1% BSA and 0.1% Tween-20 for 2 h at 25 °C. After blocking, the cells were rinsed three times with PBS, and the supernatant was then replaced with a 200 µL PBS solution containing 0.1% mouse anti-PEDV S protein monoclonal antibody (diluting 1 µL antibody in 1000 µL PBS solution containing 1% BSA and 0.1% Tween-20). The plates were kept at 37 °C for 60 min, the cells were then rinsed three times with PBS. Each of the wells was treated with 200 µL PBS solution containing Alexa Fluor 488 donkey anti-mouse IgG and DAPI (diluting 1 µL IgG and 1 µL DAPI in 1000 µL PBS solution containing 1% BSA and 0.1% Tween-20), then the plates were kept in 37 °C for 30 min. Finally, the cells were rinsed three times with PBS and the fluorescent images were captured through a fluorescence inverted microscope (Eclipse TI-U, Nikon, Tokyo, Japan).

### 2.5. Western Bolt

Cells in 12-well plates were washed three times with PBS and then incubated with 200 µL RIPA Lysis Buffer (Beyotime, Shanghai, China) at 4 °C for 10 min. The cells were scraped off and collected in 1.5 mL tubes. Then the samples were homogenized through 20 cycles of pipetting and blowing, and SDS-PAGE Sample Loading Buffer (5×) (Biosharp, Beijing, China) was added to the homogenized samples (40 µL SDS-PAGE Sample Loading Buffer (5×) in 160 µL samples). Subsequently, the samples were incubated at 100 °C for 10 min, then kept at 4 °C for 10 min. cooled samples were centrifuged at 12,000× *g* for 4 min at 4 °C. The supernatant was loaded onto SDS-PAGE gels (Epizyme Biotech, Shanghai, China) and subjected to electrophoresis, and separated proteins were transferred onto 0.2 µM polyvinyl difluoride (PVDF) membranes (Biosharp, Beijing, China). In the following step, the membranes were incubated with 5% skim milk (diluting in TBS buffer containing 0.05% Tween-20, TBST) for 2 h at room temperature, followed by TBST washes for three times. The membranes were then incubated in a solution containing 0.05% indicated antibodies (diluting 1 µL antibody in 2000 µL TBST) for 2 h at room temperature. The membranes were rinsed with TBST three times and then kept in a solution containing 0.05% HRP goat anti-mouse or HRP goat anti-rabbit antibodies for 2 h at room temperature. Finally, the membranes were washed with TBST three times and subjected to ECL Chemiluminescence substrate (Vazyme, Nanjing, China). The images were captured through a chemiluminescence detector (Tanon5200, Tanon, Shanghai, China).

### 2.6. Real-Time PCR for RNA Detection

RNA was extracted using Simply P Total RNA Extraction kit (Bioflux, Hangzhou, China) and transcribed into the corresponding cDNA through HiScript II Q RT SuperMix For qPCR (Vazyme, Nanjing, China). Hieff UNICON qPCR TaqMan Probe Master Mix (Yeasen, Shanghai, China) and Hieff UNICON qPCR SYBR Green Master Mix (Yeasen, Shanghai, China) were applied in corresponding real-time PCR tests, and all the real-time PCR was processed using QuantStudio real-time PCR system (Applied Biosystems, Waltham, MA, USA).

Primers and a probe used in this study are listed in Table 1.

### 2.7. Cellular Reactive Oxygen Species Assay

Cells were seeded to 96-well plates (black plate, black bottom) and cultured with medium containing 80 µM LA or DMSO for 24 h. Subsequently, the cells were washed three times with DMEM, then the supernatant was replaced with DMEM containing 10 µM DCFH-DA (Beyotime, Shanghai, China). The cells were kept at 37 °C with 5% CO_2_ for 1 h, then washed three times with DMEM. The values of the fluorescence signal (excitation wavelength at 488 nm and emission wavelength at 525 nm) were measured using a Multimode Plate Reader (VICTOR Nivo, PerkinElmer, Waltham, MA, USA). The ROS ratio was calculated according to the equation: Ratio = (Fs − Fb) ÷ (Fc − Fb), where Fs: values of LA treated wells; Fb: values of wells without DCFH-DA; Fc: values of DMSO-treated wells.

### 2.8. Significant Difference Analysis

GraphPad Prism6 was applied for analyzing significant differences. Differences between two groups were analyzed by using Student’s *t*-test. Asterisks indicate significant differences: * *p* < 0.05; ** *p* < 0.01; *** *p* < 0.001; **** *p* < 0.0001; ns, not significant.

## 3. Results

### 3.1. Cytotoxicity of Levistolide A

**Figure 1:**
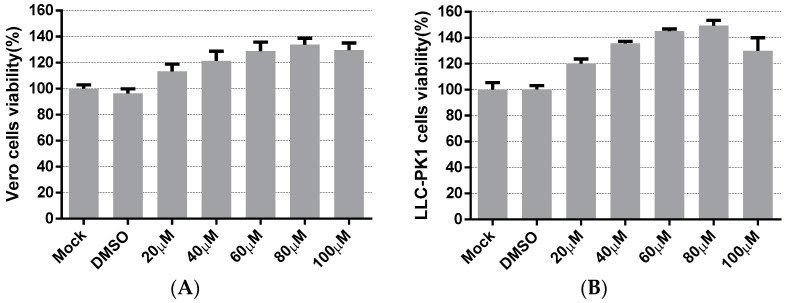
The cytotoxicity of Levistolide A on Vero and LLC-PK1 cells. Cells were treated with LA (20, 40, 60, 80, or 100 µM); DMSO served as the treatment control. The cell viability value was measured by CCK8 assay 48 h later. The value of each group was normalized by the average value (set at 100%) of the mock-treated group. (**A**) The cell viability of the Vero cells exposed to LA at designed concentrations. (**B**) The cell viability of LLC-PK1 cells exposed to LA at designed concentrations. Data represent the mean ± standard deviation (SD) of three independent experiments, and error bars represent standard deviation.

The cytotoxicity of LA on the cells was evaluated to apply an appropriate concentration of LA in subsequent experiments. Vero cells and LLC-PK1 cells were treated with LA at concentrations ranging from 20 to 100 µM. The cell viability was measured after 48 h incubation with LA, using the Cell Counting kit-8 (CCK8) assay. In Vero cells, Figure 1A indicated that the cell viability values of the LA treated groups were maintained to over 100%, compared to the mock-treated group. The cell viability values steadily climbed to the peak with the increase in the doses of LA from 20 to 80 µM, and then decreased slightly at a concentration of 100 µM. The results on LLC-PK1 cells showed cell viability to be improved after treatment with LA, and the trend was similar to the data derived from the test on Vero cells (Figure 1B). Since the cell viability values of Vero cells and LLC-PK1 cells all started to decrease at a dose of 100 µM, the maximum concentration of LA used in this study was limited to 80 µM.

### 3.2. Antiviral Activity of Levistolide A against PEDV

**Figure 2:**
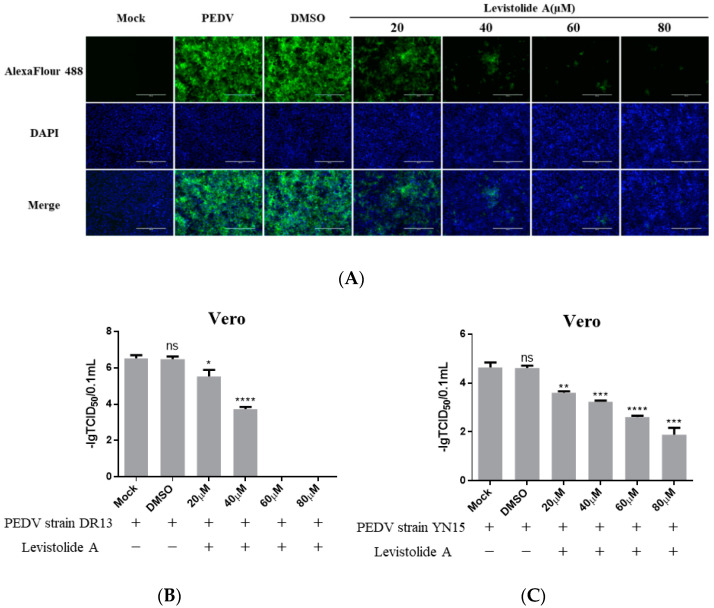

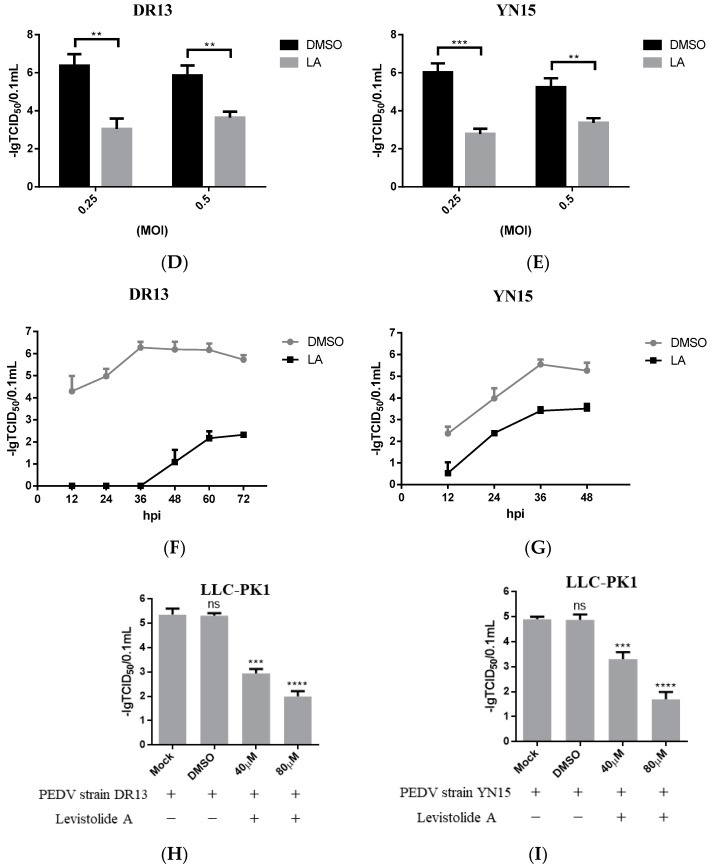
Levistolide A exhibits antiviral activity against PEDV strain DR13 and YN15 in Vero and LLC-PK1 cells. Vero cells were treated with LA at indicated concentrations and infected with PEDV at an MOI of 0.1 at the same time. DMSO served as the treatment control. Samples were collected at 36 hpi for IFA or TCID_50_ assay analysis. (**A**) Immunofluorescence of PEDV-S protein (green) detected in PEDV strain DR13 infected Vero cells. (**B**) The viral titer determined in PEDV strain DR13 infected Vero cells. (**C**) Viral titer determined in PEDV strain YN15 infected Vero cells. (**D**,**E**) Vero cells were treated with LA (80 µM) or DMSO, and exposed to PEDV at 0.25 MOI or 0.5 MOI. Viral titers were assessed at 36 hpi. (**F**,**G**) Vero cells were treated with LA (80 µM) or DMSO, and exposed to PEDV (0.1 MOI). Viral titers were assessed at designed hpi. (**H**) Viral titer determined in the PEDV strain DR13 infected LLC-PK1 cells. (**I**) The viral titer determined in PEDV strain YN15 infected LLC-PK1 cells. Viral titer level was represented by the value of −lg TCID_50_/0.1 mL. Data represent the mean ± standard deviation (SD) of three independent experiments, and error bars represent standard deviation. Asterisks indicate significant differences between mock-treated group and LA or DMSO treated group: * *p* < 0.05; ** *p* < 0.01; *** *p* < 0.001; **** *p* ≤ 0.0001; ns, not significant.

To determine whether LA possessed the ability to inhibit PEDV propagation, Vero cells were treated with LA (20, 40, 60, or 80 µM), and infected with PEDV strain DR13 at an MOI of 0.1. The cells were fixed or harvested at 36 hpi for further analyses. Immunofluorescence assay (IFA) analysis indicated that the amount of PEDV decreased significantly compared to the control groups with LA concentrations increasing from 20 µM to 80 µM (Figure 2A). The effect of LA on PEDV propagation was reconfirmed by determining the viral titer. The inhibitory effect level of LA against PEDV gradually increased with LA concentrations raising from 20 µM to 60 µM, and LA at the doses of 60 µM and 80 µM almost blocked PEDV propagation completely (Figure 2B). In order to exclude the influence of the viral strain specificity on the antiviral activity of LA against PEDV, a pandemic variant PEDV strain (strain YN15) belonging to another PEDV genogroup was applied to the same test. Figure 2C indicates that the viral titer level of LA treated groups decreased from approximately 10^4.6^ TCID_50_/0.1 mL to 10^1.89^ TCID_50_/0.1 mL while the concentrations of LA increased from 0 to 80 µM. These results illustrated LA to potently inhibit PEDV propagation in a dose-dependent manner in Vero cells. To further clarify the antiviral effect of LA against PEDV, a higher MOI of PEDV was applied in the test. Vero cells were treated with LA (80 µM) or DMSO and then exposed to PEDV at an MOI of 0.25 or 0.5. Then the viral titers were assessed at 36 hpi. In both the tests for PEDV DR13 strain and PEDV YN15 strain, although there was an increase in the initial amount of PEDV, the viral titers between the LA treatment group and the control group were significantly different (Figure 2D,E). The effect of LA on the PEDV propagation at other time points was assessed to evaluate whether the inhibitory effect of LA against PEDV was affected with time. Vero cells were treated with LA (80 µM) or DMSO and exposed to PEDV (0.1 MOI), and the samples were collected at designed hpi. When determining PEDV DR13 strain, the propagation of PEDV was found to be almost blocked in the LA treated groups before 48 hpi. Although the viral titer of the LA treated groups gradually increased after 48 hpi, the values were still lower than those in the control groups (Figure 2F). Upon assessing PEDV YN15 strain, the viral titers of the LA treated group were lower than the DMSO treated group before 48 hpi, and the growth kinetics of the LA treated group was found to reach their peak at a later time compared to the DMSO treated group (Figure 2G). These results indicated that the inhibitory effect of LA against PEDV was maintained with time elapses.

The antiviral activity of LA against PEDV was also examined in LLC-PK1 cells to eliminate the impact of cell specificity. The cells were treated with LA at a dose of 40 or 80 µM and exposed to PEDV at an MOI of 0.1, then harvested at 36 hpi for viral titer determination. The viral titers of LA treated groups were significantly lower than the mock-treated group and DMSO treated group (Figure 2H,I). These revealed that LA can also inhibit PEDV propagation in LLC-PK1 cells. In addition, the antiviral activity level increased with the rise in the LA concentrations, which was consistent with the observation in Vero cells.

### 3.3. Inhibitory Effect of Levistolide A on the Production of PEDV RNA and Protein

**Figure 3:**
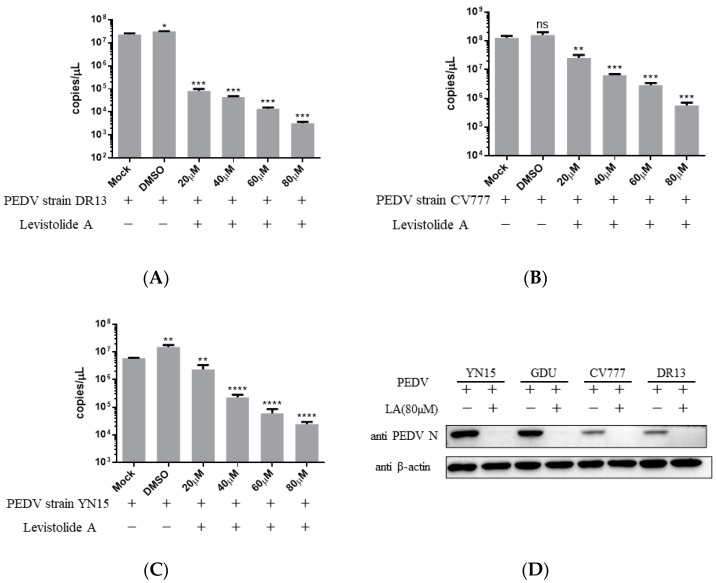
Levistolide A disturbed viral RNA and protein production in PEDV-infected cells. Vero cells were treated with LA at indicated concentrations and infected with PEDV at an MOI of 0.1 at the same time. DMSO served as the treatment control. The samples were collected at 36 hpi for TaqMan-based real-time RT-PCR or Western blot analysis. (**A**) Viral RNA copies determined in the PEDV strain DR13 infected cells; (**B**) viral RNA copies determined in PEDV strain CV777 infected cells; (**C**) viral RNA copies determined in PEDV strain YN15 infected cells. (**D**) The amount of PEDV N protein in samples was detected through Western blot; β-actin was set as the loading control. Data represent the mean ± standard deviation (SD) of three independent experiments, and error bars represent standard deviation. Asterisks indicate significant differences between the mock-treated group and LA or DMSO treated group: * *p* < 0.05; ** *p* < 0.01; *** *p* < 0.001; **** *p* ≤ 0.0001; ns, not significant.

Viral genome replication, viral mRNA transcription, and related protein translation are important for virus replication [17]. The impact of LA on PEDV RNA and protein production was evaluated to establish the relationship between the anti-PEDV activity of LA and these factors. Vero cells were treated with LA at different doses (20, 40, 60, or 80 µM) and infected with PEDV at an MOI of 0.1, then harvested at 36 hpi for TaqMan based real-time RT-PCR or Western blot analysis. The amount of PEDV RNA decreased steadily with the increase in the LA concentrations, and the trend was maintained for different PEDV strains (Figure 3A–C). Figure 3D reveals LA at a dose of 80 µM to effectively suppress the generation of PEDV N protein in PEDV-infected cells, and viral strain specificity could not disrupt this effect. The result indicated that LA could inhibit the production of PEDV RNA and proteins in PEDV-infected cells.

### 3.4. Levistolide A Exhibited an Inhibitory Effect on the PEDV-Invading Cells

**Figure 4:**
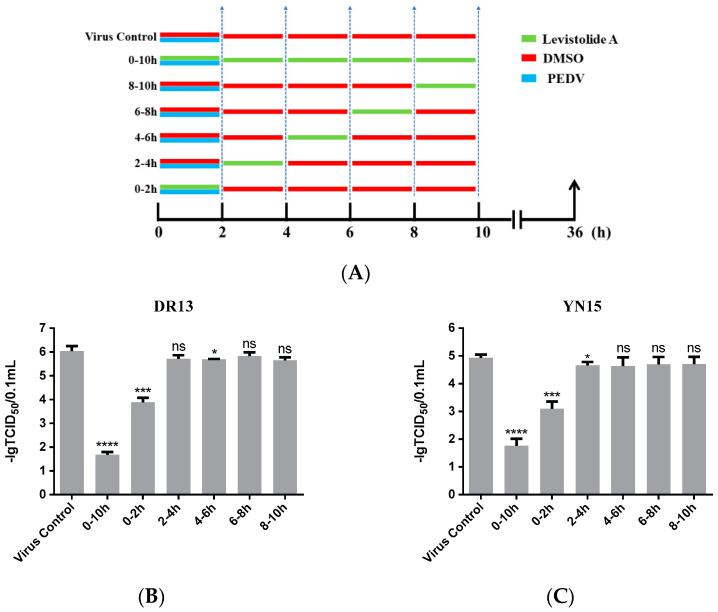
Levistolide A exhibits an inhibitory effect on the PEDV-invading Vero cells. (**A**) Vero cells were exposed to PEDV (0.1 MOI) and treated with 80 µM LA or DMSO during 0–2 hpi, then the cultured supernatant was replaced by PEDV culture medium containing LA or DMSO at 2, 4, 6, 8 hpi. The cultured supernatant in all the groups was replaced by PEDV culture medium after 10 hpi and samples were collected at 36 hpi. (**B**) The viral titer determined in the PEDV strain DR13 infected Vero cells. (**C**) The viral titer determined in the PEDV strain YN15 infected Vero cells. Data represent the mean ± standard deviation (SD) of three independent experiments, and error bars represent standard deviation. Asterisks indicate significant differences between virus control group and each LA treated group: * *p* < 0.05; ** *p* < 0.01; *** *p* < 0.001; **** *p* ≤ 0.0001; ns, not significant.

In order to reveal the precise mechanism by which LA inhibits PEDV propagation in Vero cells, the influence of LA on PEDV in different periods was assessed. As shown in Figure 4A, the cells were treated with 80 µM LA during different periods after exposure to PEDV (0.1 MOI), then harvested at 36 hpi for determining the viral titer. In this test, both strain DR13 and strain YN15 were applied in eliminating the influence of strain specificity. The results indicated that incubation with LA during 0–2 hpi effectively reduced the viral titer compared to DMSO treated group, and there was no significant change of the viral titer after treating with LA during the other periods (Figure 4B,C). The trends in the variation of the viral titer were approximately consistent between strain DR13 groups and strain YN15 groups. These illustrated that LA mainly worked during 0–2 hpi, inferring that LA might possess the potential to prevent PEDV from invading cells.

### 3.5. Anti-PEDV Activity of Levistolide A Was Associated with Inducing ROS Generation

**Figure 5:**
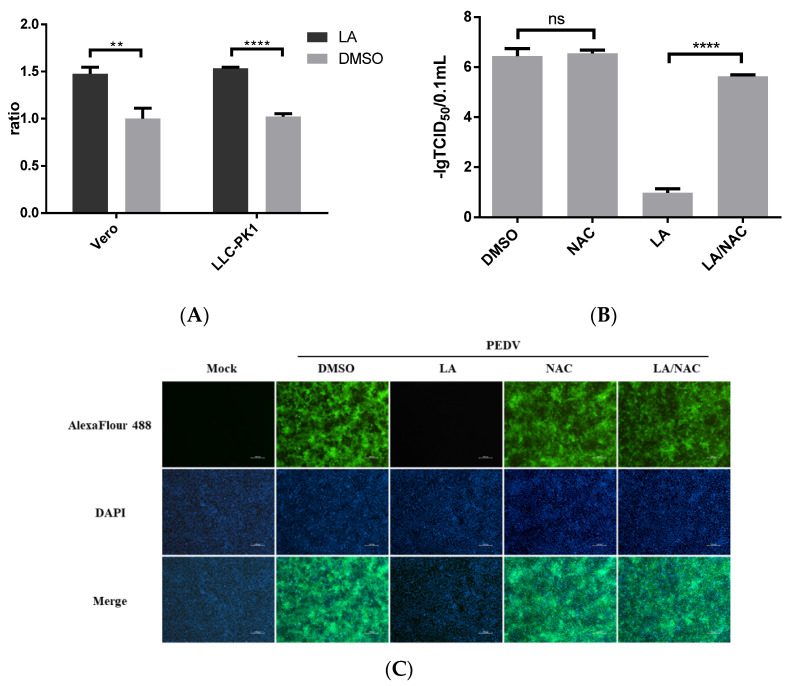
Antiviral activity of Levistolide A against PEDV is associated with inducing ROS production. (**A**) The level of ROS in Vero cells or LLC-PK1 cells after incubation with LA (80 µM) for 24 h. The value of each group was normalized by the average value (set at 1) of the corresponding DMSO treated group. (**B**) Viral titer level in LA/NAC, LA, NAC, or DMSO treated group after exposing to PEDV for 36 h. Viral titer level was represented by value of −lg TCID50/0.1 mL. (**C**) Immunofluorescence of PEDV-S protein (green) detected in LA/NAC, LA, NAC, or DMSO treated group after exposing to PEDV for 36 h. Data represent the mean ± standard deviation (SD) of three independent experiments, and error bars represent standard deviation. Asterisks indicate significant differences between virus control group and each LA treated group: * *p* < 0.05; ** *p* < 0.01; *** *p* < 0.001; **** *p* ≤ 0.0001; ns, not significant.

LA was suggested to induce ROS production [12]. We also identified the effect of LA on ROS production in Vero or LLC-PK1 cells. The cells were treated with 80 µM LA or DMSO for 24 h. The level of ROS accumulation was assessed using the Reactive Oxygen Species Assay. Figure 5A revealed that LA can promote ROS production in Vero and LLC-PK1 cells. Furthermore, ROS has been reported to be involved in regulating PEDV infection [18,19]. To clarify the role of ROS in exerting the inhibitory effect of LA against PEDV, an antioxidant, N-Acetylcysteine (NAC), was applied. Vero cells exposed to PEDV (0.1 MOI) were incubated with NAC at a dose of 5 mM and LA at a dose of 80 µM in the LA/NAC group, while the cells in other PEDV-exposed groups were treated with LA, NAC, or DMSO individually. Then, the samples were harvested at 36 hpi for further analyses. Figure 5B indicates that the titer of PEDV in the LA/NAC treated group was much larger than that in the LA alone treated group. The distance between the LA/NAC treated group and the DMSO treated group was relatively small in terms of viral titer, and incubation with NAC was unable to significantly affect PEDV propagation. The result of IFA analysis also illustrated that NAC could antagonize the inhibitory effect of LA on PEDV (Figure 5C). These demonstrated that LA might regulate PEDV replication by stimulating the level of ROS.

### 3.6. Levisrolide A Induced Endoplasmic Reticulum Stress in Vero Cells

**Figure 6:**
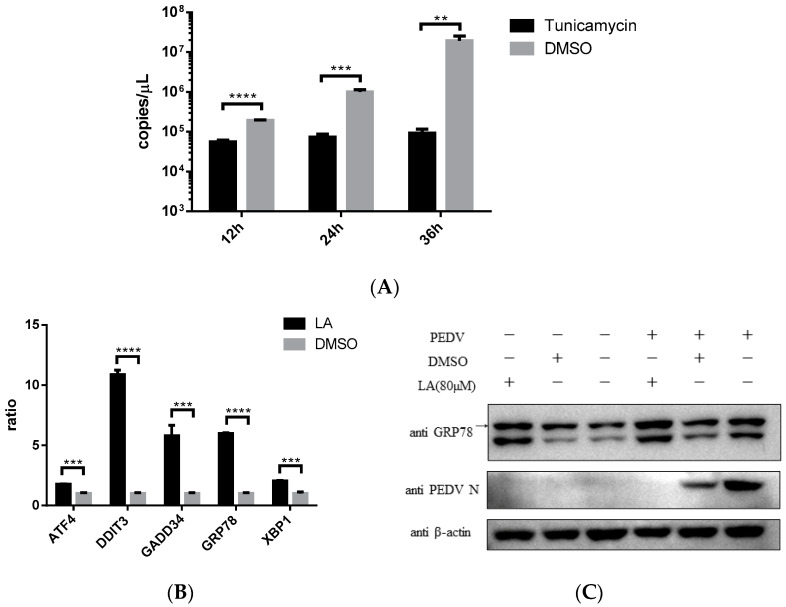
Levistolide A stimulated ER stress in Vero cells. (**A**) *Tunicamycin* (TM) potently inhibited PEDV replication. Vero cells exposed to PEDV strain YN15 (0.1 MOI) were incubated with 500 nM TM, samples were collected at 12, 24, and 36 hpi for TaqMan based real-time PCR; (**B**) mRNA level of ER stress-related genes increased in LA Vero cells. (**C**) The amount of GRP78 increased by treating with LA in either PEDV or mock infected Vero cells. PEDV strain YN15 (0.1 MOI) or mock infected Vero cells were incubated with LA or DMSO for 36 hpi. Western blot was performed to assess protein expression level and β-actin was set as loading control. Bands for GRP78 were marked by a black arrow. Data represent the mean ± standard deviation (SD) of three independent experiments, and error bars represent standard deviation. Asterisks indicate significant differences between virus control group and each LA treated group: * *p* < 0.05; ** *p* < 0.01; *** *p* < 0.001; **** *p* ≤ 0.0001; ns, not significant.

Endoplasmic reticulum stress (ER stress) was reported to be associated with ROS increase in cells [20]. Stimulating ER stress was illustrated to be able to inhibit PEDV propagation [21]. This study also indicated that ER stress agonist, *Tunicamycin*, can effectively restrict PEDV strain YN15 replication in Vero cells (Figure 6A). According to these, the effect of LA on ER stress was assessed. Vero cells were incubated with 80 µM LA or DMSO for 24 h, then the samples were processed for SYBR based real-time PCR. Figure 6B indicated that the mRNA level of ER stress-related genes (*ATF4*, *DDIT3*, *GADD34*, *GRP78*, and *XBP1*) were all upregulated, demonstrating that LA can stimulate ER stress. The result of Western blot analysis reconfirmed this declaration. The amount of GRP78, a marker protein of ER stress, was increased under LA treatment in either PEDV or mock infected Vero cells (Figure 6C). Since simulating ER stress can inhibit PEDV replication, and ER stress was an important consequence of ROS generation in PEDV-infected cells [12,18], LA might suppress PEDV propagation via ROS-mediated ER stress.

## 4. Discussion

PEDV is a hazardous pathogen causing fatal diarrhea in piglets posing a severe adverse impact on global pig production. There are differences in sequence between the traditional and variant strains of PEDV [22]. The vaccine-derived from the PEDV traditional strain imparts negligible protection against the variant [7,23]. Sequence diversity has also been shown to be complicated among different PEDV variant strains and their antigenicity might possess variation [24]. This necessitates the establishment of alternative strategies for defending PEDV infection apart from vaccine development. Developing antiviral agents has been considered an effective way of controlling the corresponding disease. For instance, antiviral therapy appears to be an attractive approach for controlling African swine fever virus infection, which did not have any applicable vaccine at the time [25]. The development of effective antiviral agents against PEDV has been eagerly investigated and studies have revealed that some molecules possess the potential to control PEDV [21,26,27,28,29], but there is still no applicable drug in clinical practice [28].

This is the first study reporting Levistolide A, a natural product, to possess antiviral activity against PEDV. Levistolide A has been described to effectively inhibit the proliferation of cancer cells [12,13]. LA was also reported to be a candidate for treating Alzheimer’s disease [14]. Moreover, LA has been indicated for defending against infectious diseases [15]. In this study, LA was indicated for suppressing PEDV propagation in vitro. The treatment with LA showed potent inhibition against PEDV in both Vero cells and a porcine cell line, the LLC-PK1 cell. Inducing ROS generation was proven to play a key part in exerting this inhibitory effect.

PEDV strains can be sorted into two genogroups, GI (classical) and GII (variant). Each genogroup can then be divided into two sub-groups, GI-a, GI-b, GII-a, and GII-b [5,6,9,30]. The rate of PEDV strain belonging to GII genogroup has been revealed to escalate in China between 2011 to 2018 and the strains of the GII-b sub-group seemed to gradually occupy a dominant position [5,22,31,32]. Besides China, the strains of the GII-b sub-group have been reported to circulate in other Asian countries including Thailand, Vietnam, Japan, and South Korea [9,33,34]. The GII-b sub-group strains have also been identified in the USA [35]. The development of antiviral drugs for GII-b sub-group strains is highly significant. In this study, the effect of LA on four PEDV strains has been evaluated. Among them, both DR13 and CV777 belong to the G1 genogroup, while YN15 and GDU belong to the GII-b group. LA showed a potent inhibitory effect on both strain DR13 and strain YN15, and LA can suppress N protein production in terms of these four strains. These indicated that LA can potentially defend these PEDV strains. However, the effect of LA on the strains belonging to GII-a sub-group needs further examination to reveal the antiviral activity of LA against PEDV.

Attachment and entry are important processes in the life cycle of different virus [36,37,38,39,40]. Moreover, these processes can also be key points in the propagation of coronavirus [17,41]. Inhibitors targeting these two processes are likely to assist in developing antiviral drugs [37,42]. For instance, the methanolic extracts of *Hybanthus enneaspermus* suppressed HBV by inhibiting the binding of HBs to Ag [43]. Quercetin inhibited the propagation of a variety of viruses by blocking viruses attaching to the cellular membrane or penetrating the cells [44,45,46]. Griffithsin suppressed HIV by binding to HIV envelope protein gp120, which is important for cellular entry of HIV [47]. There were also several agents shown to exert antiviral effects against PEDV by affecting viral attachment and entry. Carbazole derivatives have been indicated to suppress PEDV replication by interfering with viral attachment [28]. Quercetin 7-rhamnoside mainly exerted antiviral activity during the period for attachment and entry [48]. Moreover, Salinomycin was also documented to affect PEDV penetrating Vero cells [49]. This study illustrated LA to mainly inhibit PEDV during 0–2 hpi, while this period has been defined as the main stage for the attachment and entry of PEDV [50,51]. This establishes that LA might be able to interact with proteins on the cell surface or viral surface. It is noteworthy to reveal the mechanism of this observation and might help explore cell receptors for PEDV since the main cell receptor for PEDV is still unknown [52,53].

Reactive oxygen species (ROS) participate in regulating various physiological functions of cells [54]. Reports have indicated that ROS are involved in signal transmission in cells [55]. ROS at moderate levels can benefit cells in terms of cellular proliferation, metabolic regulation, physiological, and pathogen defense [56]. ROS have also been discussed to be closely related to viruses. ROS have played a role in virus propagation, including SARS-CoV2, Hepatitis B virus, and Epstein–Barr virus [57,58,59], and proteins derived from viruses, such as African swine fever virus and Influenza virus, were reported to participate in regulating ROS generation [60,61]. The ROS level was also revealed to increase in PEDV-infected cells [18]. As the antioxidants were inactive against PEDV infection [27], upregulating the ROS level can serve as a defense mechanism in mitigating PEDV infection. In this study, LA was indicated to stimulate ROS generation, and inhibiting ROS production could antagonize the anti-PEDV activity of LA. It illustrated that inducing ROS was a key event that resulted in LA inhibiting PEDV propagation. ER stress is an important consequence of ROS generation, and can effectively inhibit PEDV replication [18,21]. We stated that LA can upregulate the mRNA level of ER stress-related genes and can stimulate the expression of the ER stress-marker protein. It is possible that LA inhibited PEDV propagation via ROS-mediated ER stress.

In conclusion, we declared LA can potently inhibit PEDV propagation in vitro, and this effect was possibly associated with ROS-mediated ER stress. This study can therefore provide the theoretical support for developing anti-PEDV drug development.

## Figures and Tables

**Table 1:** Primers and a probe used in this study.

Primer	Sequence (5′–3′)
PEDV F	CGTACAGGTAAGTCAATTAC
PEDV R	GATGAAGCATTGACTGAA
PEDV probe-M	FAM-TTCGTCACAGTCGCCAAGG-TAMRA
ACTB-F	CTACACCGCTACCAGTTCGC
ACTB-R	TAGGAGTCCTTCTGGCCCAT
GADD34-F	GTCAGGACCTGTGATCGCTT
GADD34-R	CCATGTGTCTGGGCGGTG
ATF4-F	CCAACAACGGCAAGGAGGATG
ATF4-R	GGGCATCAAAGTCGAACTCC
GRP78-F	CTTCGGCGTGAGGTGGAAAA
GRP78-R	ACCGGAACAGATCCATGTTGA
DDIT3-F	GGAACCTGAGGAGAGAGTGTTC
DDIT3-R	CTGCCATCTCTGCAGTTGGA
B2M-F	GCGGAATATAAGTGGAGGCGT
B2M-R	ATCTTTGGAGCACGCTGGATA
XBP1-F	CGGCCTTGTAGTTGAGAACCA
XBP1-R	TCACTCCATTCCCCTTGGCTT
TGEV-F	ACGCCGTATAGTGCAGATGG
TGEV-R	AGTTGCACTTTTGCCGCATT
PDCoV-F	AAGGATGCGCTCAATACGGT
PDCoV-R	TGTCTGCTGGCAGAGTTACC

## References

[1] Lv C., Xiao Y., Li X., Tian K. (2016). Porcine epidemic diarrhea virus: Current insights. Virus Adapt. Treat..

[2] Jung K., Saif L.J., Wang Q. (2020). Porcine epidemic diarrhea virus (PEDV): An update on etiology, transmission, pathogenesis, and prevention and control. Virus Res..

[3] Li Z., Ma Z., Li Y., Gao S., Xiao S. (2020). Porcine epidemic diarrhea virus: Molecular mechanisms of attenuation and vaccines. Microb. Pathog..

[4] Song D., Park B. (2012). Porcine epidemic diarrhoea virus: A comprehensive review of molecular epidemiology, diagnosis, and vaccines. Virus Genes.

[5] Su M., Li C., Qi S., Yang D., Sun D. (2019). A molecular epidemiological investigation of PEDV in China: Characterization of co-infection and genetic diversity of S1-based genes. Transbound. Emerg. Dis..

[6] Wang D., Fang L., Xiao S. (2016). Porcine epidemic diarrhea in China. Virus Res..

[7] Sun R.Q., Cai R.J., Chen Y.Q., Liang P.S., Song C.X. (2012). Outbreak of Porcine Epidemic Diarrhea in Suckling Piglets, China. Emerg. Infect. Dis..

[8] Langel S.N., Paim F.C., Lager K.M., Vlasova A.N., Saif L.J. (2016). Lactogenic immunity and vaccines for porcine epidemic diarrhea virus (PEDV): Historical and current concepts. Virus Res..

[9] Lee C. (2015). Porcine epidemic diarrhea virus: An emerging and re-emerging epizootic swine virus. Virol. J..

[10] He W.-Q., Lv W.-S., Zhang Y., Qu Z., Wei R.-R., Zhang L., Liu C.H., Zhou X.X., Li W.R., Huang X.T. (2015). Study on Pharmacokinetics of Three Preparations from Levistolide A by LC-MS-MS. J. Chromatogr. Sci..

[11] Chen F., Wang T., Wang J., Wang Z.-Q., Qian M. (2008). Levistolide A overcomes P-glycoprotein-mediated drug resistance in human breast carcinoma cells. Acta Pharmacol. Sin..

[12] Yang Y., Zhang Y., Wang L., Lee S. (2017). Levistolide A Induces Apoptosis via ROS-Mediated ER Stress Pathway in Colon Cancer Cells. Cell. Physiol. Biochem..

[13] Ding Y., Niu W., Zhang T., Wang J., Cao J., Chen H., Wang R., An H. (2018). Levistolide A synergistically enhances doxorubicin induced apoptosis of k562/dox cells by decreasing MDR1 expression through the ubiquitin pathway. Oncol. Rep..

[14] Qu X., Guan P., Han L., Wang Z., Huang X. (2021). Levistolide A Attenuates Alzheimer’s Pathology Through Activation of the PPARgamma Pathway. Neurother. J. Am. Soc. Exp. NeuroTher..

[15] Beck J.J., Stermitz F.R. (1995). Addition of methyl thioglycolate and benzylamine to (Z)-ligustilide, a bioactive unsaturated lactone constituent of several herbal medicines. An improved synthesis of (Z)-ligustilide. J. Nat. Prod..

[16] Li W., Zhang M., Zheng H., Zhou P., Liu Z., Jongkaewwattana A., Luo R., He Q. (2021). Construction of a Recombinant Porcine Epidemic Diarrhea Virus Encoding Nanoluciferase for High-Throughput Screening of Natural Antiviral Products. Viruses.

[17] Fung T.S., Liu D.X. (2019). Human Coronavirus: Host-Pathogen Interaction. Annu. Rev. Microbiol..

[18] Sun P., Jin J., Wang L., Wang J., Xu X. (2020). Porcine epidemic diarrhea virus infections induce autophagy in Vero cells via ROS-dependent endoplasmic reticulum stress through PERK and IRE1 pathways. Vet. Microbiol..

[19] Xu X., Xu Y., Zhang Q., Yang F., Yin Z., Wang L., Li Q. (2019). Porcine epidemic diarrhea virus infections induce apoptosis in Vero cells via a reactive oxygen species (ROS)/p53, but not p38 MAPK and SAPK/JNK signalling pathways. Vet. Microbiol..

[20] Hafiz Z., Geum L., Hyung-Ryong K., Han-Jung C. (2016). Endoplasmic Reticulum Stress and Associated ROS. Int. J. Mol. Sci..

[21] Wang Y., Li J.R., Sun M.X., Ni B., Huan C., Huang L., Li C., Fan H.J., Ren X.F., Mao X. (2014). Triggering unfolded protein response by 2-Deoxy-D-glucose inhibits porcine epidemic diarrhea virus propagation. Antivir. Res..

[22] Zhu T., Du S., Cao D., Pei Z., Guo Y., Shao H., Wang H., Wang K., Hu G. (2019). Isolation and identification of a variant subtype G 2b porcine epidemic diarrhea virus and S gene sequence characteristic. Infect. Genet. Evol. J. Mol. Epidemiol. Evol. Genet. Infect. Dis..

[23] Wang J., Zhao P., Guo L., Liu Y., Du Y., Ren S., Li J., Zhang Y., Fan Y., Huang B. (2013). Porcine Epidemic Diarrhea Virus Variants with High Pathogenicity, China. Emerg. Infect. Dis..

[24] Lin C.M., Saif L.J., Marthaler D., Wang Q. (2016). Evolution, antigenicity and pathogenicity of global porcine epidemic diarrhea virus strains. Virus Res..

[25] Arabyan E., Kotsynyan A., Hakobyan A., Zakaryan H. (2019). Antiviral agents against African swine fever virus. Virus Res..

[26] Chen Y., Luo Q., Li S., Li C., Yang Y. (2020). Antiviral activity against porcine epidemic diarrhea virus of Pogostemon cablin polysaccharide. J. Ethnopharmacol..

[27] Song J., Shim J., Choi H. (2011). Quercetin 7-rhamnoside reduces porcine epidemic diarrhea virus replication via independent pathway of viral induced reactive oxygen species. Virol. J..

[28] Chen Z., Chen J., Wei X., Hua H., Hu R., Ding N., Zhang J., Song D., Ye Y., Tang Y. (2021). Antiviral Activities of Carbazole Derivatives against Porcine Epidemic Diarrhea Virus In Vitro. Viruses.

[29] Li Z., Cao H., Cheng Y., Zhang X., Han H. (2020). Inhibition of Porcine Epidemic Diarrhea Virus Replication and Viral 3C-Like Protease by Quercetin. Int. J. Mol. Sci..

[30] Li D., Li Y., Liu Y., Chen Y., Zhang G. (2021). Isolation and Identification of a Recombinant Porcine Epidemic Diarrhea Virus With a Novel Insertion in S1 Domain. Front. Microbiol..

[31] Jing S., Qunjing L., Chunyan S., Yuanmei M., Haijian H., Sheng J., Yingshan Z., Yuan W., Shaobo B., Lin S. (2018). Isolation and characterization of Chinese porcine epidemic diarrhea virus with novel mutations and deletions in the S gene. Vet. Microbiol..

[32] Huan C., Pan H., Fu S., Xu W., Liu X. (2020). Characterization and evolution of the coronavirus porcine epidemic diarrhoea virus HLJBY isolated in China. Transbound. Emerg. Dis..

[33] Temeeyasen G., Srijangwad A., Tripipat T., Tipsombatboon P., Piriyapongsa J., Phoolcharoen W., Chuanasa T., Tantituvanont A., Nilubol D. (2014). Genetic diversity of ORF3 and spike genes of porcine epidemic diarrhea virus in Thailand. Infect. Genet. Evol..

[34] Vui D.T., Tung N., Inui K., Slater S., Nilubol D. (2014). Complete Genome Sequence of Porcine Epidemic Diarrhea Virus in Vietnam. Genome Announc..

[35] Huang Y.W., Dickerman A.W., Pieyro P., Li L., Meng X.J. (2013). Origin, Evolution, and Genotyping of Emergent Porcine Epidemic Diarrhea Virus Strains in the United States. Mbio.

[36] Guo Y., Duan M., Wang X., Gao J., Guan Z., Zhang M. (2019). Early Events in Rabies Virus Infection—Attachment, Entry, and Intracellular Trafficking. Virus Res..

[37] Ming L. (2012). Influenza virus entry. Adv. Exp. Med. Biol..

[38] Graziano V.R., Wei J., Wilen C.B. (2019). Norovirus Attachment and Entry. Viruses.

[39] Zeisel M.B., Felmlee D.J., Baumert T.F. (2013). Hepatitis C Virus Entry. J. Biol. Chem..

[40] Koehler M., Delguste M., Sieben C., Gillet L., Alsteens D. (2020). Initial Step of Virus Entry: Virion Binding to Cell-Surface Glycans. Annu. Rev. Virol..

[41] Fehr A.R., Perlman S. (2015). Coronaviruses: An Overview of Their Replication and Pathogenesis. Methods Mol. Biol..

[42] Altmeyer R. (2004). Virus Attachment and Entry Offer Numerous Targets for Antiviral Therapy. Curr. Pharm. Des..

[43] Muruganantham S. (2015). In vitro screening of anti-hbv properties of selected indian medicinal plants from kolli hills, namakkal district of tamilnadu, india. World J. Pharm. Pharm. Sci..

[44] Wu W., Li R., Li X., He J., Jiang S., Liu S., Yang J. (2016). Quercetin as an Antiviral Agent Inhibits Influenza A Virus (IAV) Entry. Viruses.

[45] Lopes B., da Costa M.F., Genova Ribeiro A., da Silva T.F., Lima C.S., Caruso I.P., de Araujo G.C., Kubo L.H., Iacovelli F., Falconi M. (2020). Quercetin pentaacetate inhibits in vitro human respiratory syncytial virus adhesion. Virus Res..

[46] Sun Y., Li C., Li Z., Shangguan A., Jiang J., Zeng W., Zhang S., He Q. (2021). Quercetin as an antiviral agent inhibits the Pseudorabies virus in vitro and in vivo. Virus Res..

[47] Sabrina L., Carole B. (2016). Griffithsin: An Antiviral Lectin with Outstanding Therapeutic Potential. Viruses.

[48] Choi H.-J., Kim J.-H., Lee C.-H., Ahn Y.-J., Song J.-H., Baek S.-H., Kwon D.-H. (2009). Antiviral activity of quercetin 7-rhamnoside against porcine epidemic diarrhea virus. Antivir. Res..

[49] Yuan C., Huang X., Zhai R., Ma Y., Xu A., Zhang P., Yang Q. (2021). In Vitro Antiviral Activities of Salinomycin on Porcine Epidemic Diarrhea Virus. Viruses.

[50] Tong T., Hu H., Zhou J., Deng S., Zhang X., Tang W., Fang L., Xiao S., Liang J. (2020). Glycyrrhizic-Acid-Based Carbon Dots with High Antiviral Activity by Multisite Inhibition Mechanisms. Small.

[51] Wang P., Bai J., Liu X., Wang M., Wang X., Jiang P. (2020). Tomatidine inhibits porcine epidemic diarrhea virus replication by targeting 3CL protease. Vet. Res..

[52] Li W., Luo R., He Q., Kuppeveld F.V., Rottier P., Bosch B.J. (2017). Aminopeptidase N is not required for porcine epidemic diarrhea virus cell entry. Virus Res..

[53] Ji C.-M., Wang B., Zhou J., Huang Y.-W. (2018). Aminopeptidase-N-independent entry of porcine epidemic diarrhea virus into Vero or porcine small intestine epithelial cells. Virology.

[54] Yang B., Chen Y., Shi J. (2019). Reactive Oxygen Species (ROS)-Based Nanomedicine. Chem. Rev..

[55] D’Autréaux B., Toledano M.B. (2007). ROS as signalling molecules: Mechanisms that generate specificity in ROS homeostasis. Nat. Rev. Mol. Cell Biol..

[56] Ron M. (2016). ROS Are Good. Trends Plant Sci..

[57] Arcanjo A., Logullo J., Menezes C., Giangiarulo T., Morrot A. (2020). The Emerging Role of Neutrophil Extracellular Traps in Severe Acute Respiratory Syndrome Coronavirus 2 (COVID-19). Sci. Rep..

[58] Montani M.G., Santarelli R., Granato M., Gonnella R., Torrisi M.R., Faggioni A., Cirone M. (2018). EBV reduces autophagy, intracellular ROS and mitochondria to impair monocyte survival and differentiation. Autophagy.

[59] Yu X., Lan P., Hou X., Han Q., Lu N., Li T., Jiao C., Zhang J., Zhang C., Tian Z. (2017). HBV inhibits LPS-induced NLRP3 inflammasome activation and IL-1β production via suppressing the NF-kappa B pathway and ROS production. J. Hepatol. J. Eur. Assoc. Study Liver.

[60] Xia N., Wang H., Liu X., Shao Q., Zhu J. (2020). African Swine Fever Virus Structural Protein p17 Inhibits Cell Proliferation through ER Stress—ROS Mediated Cell Cycle Arrest. Viruses.

[61] Wang R., Zhu Y., Lin X., Ren C., Zhao J., Wang F., Gao X., Xiao R., Zhao L., Chen H. (2019). Influenza M2 protein regulates MAVS-mediated signaling pathway through interacting with MAVS and increasing ROS production. Autophagy.

